# Uranium concentration in blood samples of Southern Iraqi leukemia patients using CR-39 track detector

**DOI:** 10.1007/s10967-013-2808-0

**Published:** 2013-11-05

**Authors:** Anees A. Al-Hamzawi, M. S. Jaafar, Nada F. Tawfiq

**Affiliations:** 1 School of Physics, Universiti Sains Malaysia, 11800 Penang, Malaysia; 2 Department of Physics, College of Education, Al-Qadisiyah University, Qadisiyah, Iraq; 3 Department of Physics, College of Science, Al-Nahrain University, Baghdad, Iraq

**Keywords:** Uranium concentration, Leukemia, CR-39, SSNTDs, Human blood, Southern Iraq

## Abstract

The simple and effective technique of fission track etch has been applied to determine trace concentration of uranium in human blood samples taken from two groups of male and female participants: leukemia patients and healthy subjects group. The blood samples of leukemia patients and healthy subjects were collected from three key southern governorates namely, Basrah, Muthanna and Dhi-Qar. These governorates were the centers of intensive military activities during the 1991 and 2003 Gulf wars, and the discarded weapons are still lying around in these regions. CR-39 track detector was used for registration of induced fission tracks. The results show that the highest recorded uranium concentration in the blood samples of leukemia patients was 4.71 ppb (female, 45 years old, from Basrah) and the minimum concentration was 1.91 ppb (male, 3 years old, from Muthanna). For healthy group, the maximum uranium concentration was 2.15 ppb (female, 55 years old, from Basrah) and the minimum concentration was 0.86 ppb (male, 5 years old, from Dhi-Qar). It has been found that the uranium concentrations in human blood samples of leukemia patients are higher than those of the healthy group. These uranium concentrations in the leukemia patients group were significantly different (*P* < 0.001) from those in the healthy group.

## Introduction

Uranium is one of the most serious contamination concerns because of its radioactivity and heavy-metal toxicity. Uranium and its compounds are highly toxic, which is a threat to human health and ecological balance [1]. Uranium is widespread in nature, and it exists in the form of solid, liquid, and gaseous compounds. It readily combines with other elements to form uranium oxide, silicates, carbonates, and hydroxides [2]. Uranium is used as fuel in nuclear power plants and is present, in the different steps of the nuclear industry, in different forms with different isotopic compositions (natural, depleted, and enriched). Depleted uranium (DU) is a byproduct of the nuclear industry. Its specific activity is approximately 40 % lower than that of naturally occurring uranium. Because of its high density and metallurgical properties, DU is used in the manufacture of armor and armor piercing shells in several countries [3, 4]. The first use of DU was in the Gulf war in 1991 [5]. In the south of Iraq, DU was and still an environmental pollution problem because its levels raised after both Gulf wars I and II in 1991 and 2003 respectively [6].

There are different possible ways by which uranium can reach the human body either in a direct way by inhaling uranium-bearing dust particles or by drinking water which is polluted by uranium, or in an indirect way from the fertile soil layer via the food chain [7]. Solubility of uranium varies depending on the particular compounds and the solvent, and this solubility determines how quickly and efficiently the body absorbs them through the lungs and the intestines, respectively [8].Uranium deposited in the bones and other organs is subsequently released back into the blood stream, which causes several health problems ranging from cancer to kidney failure, leukemia, respiratory disorders, congenital abnormalities, skin diseases, and other obscure unknown diseases [9–11].

Researchers at the Armed Forces Radiobiology Research Institute (AFRRI) in Bethesda and others have found that uranium causes mutations in DNA [12, 13] and uranium exposure can result in increased chromosomal aberrations [14–16]. It is a widely accepted principle in molecular biology that agents that cause mutations or damage DNA can cause cancer.

In Iraq, the incidence of cancers that are registered annually by Iraqi Cancer Board involved an increase in the number of cancer cases that are recorded after the Gulf wars [17].

There are some areas in the southern of Iraq like (Basrah, Muthanna and Dhi-Qar) that have experienced a twofold to fivefold increase in reported cancers. Most of these cases involve damage to the lungs, bronchial tubes, bladder, and skin. In addition, an increased incidence of stomach cancer in males and breast cancer in females has also been reported, as well as an overall increase in leukemia cases [18].

Solid-state nuclear track detectors (SSNTDs) are normally used to determine the uranium concentration in human blood [10, 11]. The fission track technique was suggested by Fleischer et al. [19] who proposed the method of using thermal neutron irradiation of solid-state track-recording materials that are in contact with both films and pressed together to dry the blood. This technique appears particularly suitable for quantitative determination of uranium in the blood.

The aim of this study is to determine the uranium concentration in the blood of the leukemia patients and the healthy group using CR-39 nuclear track detector.

## Material and method

### Sample collection

**Fig. 1:**
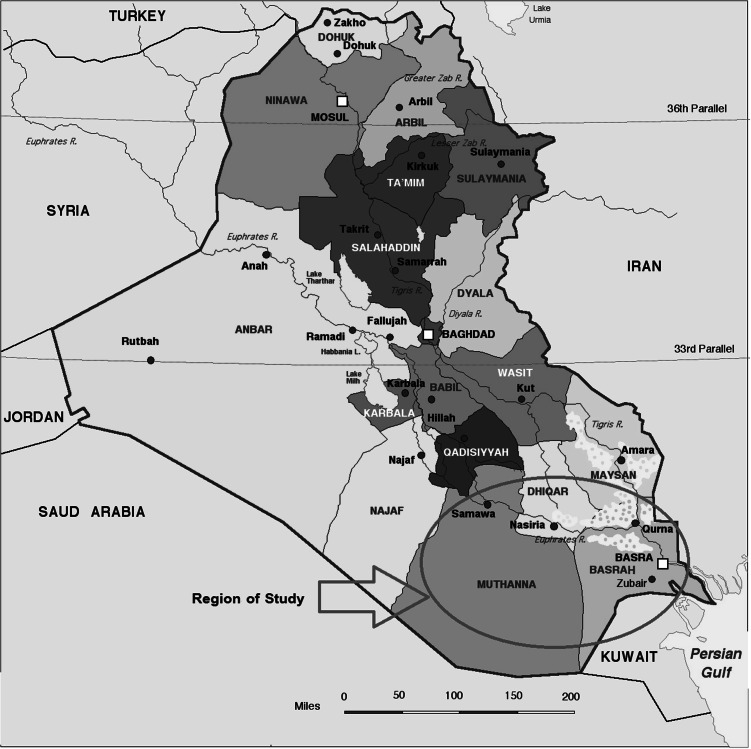
The location of the three governorates (Basrah, Muthanna and Dhi-Qar) involved in the study

In this study, 60 blood samples of volunteers, males and females, were collected from two groups. The first group included the leukemia patients by which 30 blood samples were collected from hospitals in Basrah, Muthanna and Dhi-Qar. While the second group involved the healthy volunteers and the samples were gathered from 30 healthy volunteers who live in these governorates in the southern of Iraq Fig. 1. The volunteers from these groups had no previous history of occupational exposure to uranium. They completed a comprehensive questionnaire about demographic information such as age, gender, and medical history. The ratio between the genders for these groups was more balanced Table 1.

**Table 1:** Descriptive statistics of the two groups

	Leukemia patients group	Healthy group
Number of males	14	14
Number of females	16	16
Age range/years	2–71	3–57
Average age/years (Males)	20.64	20.71
Average age/years (Females)	21.43	19.37
Average age/years (Total)	21.1	20

## Experimental method

The experimental technique for the investigation of uranium concentration is the same as reported elsewhere [10, 11, 21, 22].

Blood samples were heated at 37 °C for 24 h using an electric heating incubator to dry and to oxidize organic material. The powders collected in the form of 0.5 g of dried powder blood were mixed with 0.1 g of methylcellulose (C6 H10 O5) used as a binder. The mixture was pressed into a pellet of 1 cm diameter and 1.5 mm thickness. The pellets were covered with CR-39 track detector on both sides and were put in a plate of paraffin wax at a distance of 5 cm from Am–Be neutron source with a thermal flounce equal to (3.024 × 10^9^ n cm^−2^) for 7 days, to cause latent damage to the detector due to ^235^U (n, f) reaction.

After the irradiation, the CR-39 detectors were etched in (*N* = 6.25) NaOH solution at temperature of 60 °C for 5 h. The induced fission tracks densities were recorded using Olympus optical microscope with magnification of 400× . The fission track densities were measured on the surfaces, showing uniform distribution of uranium.

### Calculations

The investigation into the uranium concentration in the blood samples was carried out by comparing between the densities of track registered on CR-39 detectors around the sample pellet and that of standard samples pellet, via the following relation [10, 23]:

1 $$ U_{\text{x}} = \, U_{\text{s}} \rho_{\text{x}} / \, \rho_{\text{s}} . \, I_{\text{s}} / I_{\text{x}} . \, R_{\text{s}} / \, R_{\text{x}} $$

Where:

*U*_x_ and *U*_s_ are the uranium concentration for the unknown and the standard samples.

*ρ*_x_ and *ρ*_s_ are the densities of fission tracks for the unknown and the standard samples.

*I*_x_ and *I*_s_ are the isotopic abundance ratio of ^235^U to ^238^U for the unknown and standard samples.

*R*_x_ and *R*_s_ are the range of fission fragments in mg cm^−2^ for the unknown and standard samples.

The correction factor (*R*_s_ */R*_x_ *)* is taken to be unity. Similarly, taking *(I*_s_ */I*_x_ *)* as unity, the equation becomes

2 $$ U_{\text{x}} = U_{\text{s}} \rho_{\text{x}} / \, \rho_{\text{s}} $$

### Statistical analysis

All the results that are obtained from all samples of the two groups were statistically analyzed using Statistical Package of the Social Sciences (SPSS) and the significance of the probability level (*P*) was estimated by Independent sample *t* Test.

## Result and discussion

Table 2 shows the uranium concentration in blood samples of the leukemia patients group. The maximum value obtained was 4.71 ppb which belongs to a female (45 years from Basrah), and the minimum value of the leukemia patients group is 1.91 ppb for a male child (3 years from Muthanna). The mean value of uranium concentration of this group is 2.87 ppb.

**Table 2:** Uranium concentration in blood samples of the leukemia patients group

Sample	Gender	Age (Year)	Location	Uranium concentration in (ppb) ± S.D.
1	Male	7	Basrah	2.89 ± 0.18
2	Female	6	Basrah	3.06 ± 0.21
3	Male	71	Basrah	3.11 ± 0.19
4	Female	2	Basrah	2.69 ± 0.24
5	Female	52	Basrah	3.25 ± 0.13
6	Male	7	Basrah	3.09 ± 0.21
7	Male	6	Basrah	3.04 ± 0.24
8	Male	62	Basrah	3.18 ± 0.22
9	Female	29	Basrah	3.15 ± 0.17
10	Female	45	Basrah	4.71 ± 0.17
11	Female	8	Muthanna	2.62 ± 0.21
12	Male	3	Muthanna	1.91 ± 0.24
13	Male	17	Muthanna	2.73 ± 0.20
14	Female	2	Muthanna	2.54 ± 0.21
15	Male	5	Muthanna	2.33 ± 0.13
16	Male	17	Muthanna	2.27 ± 0.12
17	Female	15	Muthanna	3.04 ± 0.11
18	Female	28	Muthanna	2.92 ± 0.18
19	Male	16	Muthanna	4.68 ± 0.17
20	Female	50	Muthanna	2.71 ± 0.15
21	Male	24	Dhi-Qar	2.22 ± 0.23
22	Female	59	Dhi-Qar	2.89 ± 0.13
23	Female	7	Dhi-Qar	3.03 ± 0.21
24	Male	15	Dhi-Qar	2.97 ± 0.25
25	Female	8	Dhi-Qar	1.98 ± 0.14
26	Female	12	Dhi-Qar	2.84 ± 0.18
27	Female	8	Dhi-Qar	2.43 ± 0.18
28	Male	11	Dhi-Qar	2.7 ± 0.18
29	Male	50	Dhi-Qar	2.45 ± 0.16
30	Female	60	Dhi-Qar	2.61 ± 0.15
Mean ± Std Error				2.87 ± 0.11

Table 3 shows the uranium concentration in blood samples of the healthy group. The maximum value obtained was 2.15 ppb which belongs to a female (55 years from Basrah), and the minimum value of the healthy group is 0.86 ppb for a male child (5 years from Dhi-Qar).The mean value of uranium concentration of this group is 1.43 ppb.

**Table 3:** Uranium concentration in blood samples of the healthy group

Sample	Gender	Age (year)	Location	Uranium concentration in (ppb) ± S.D.
1	Female	6	Basrah	1.41 ± 0.14
2	Male	4	Basrah	0.91 ± 0.11
3	Male	10	Basrah	0.96 ± 0.1
4	Female	15	Basrah	1.77 ± 0.14
5	Male	20	Basrah	1.73 ± 0.15
6	Male	57	Basrah	2.1 ± 0.14
7	Male	32	Basrah	2.07 ± 0.2
8	Female	55	Basrah	2.15 ± 0.25
9	Female	3	Basrah	0.92 ± 0.11
10	Female	5	Basrah	1.28 ± 0.14
11	Female	10	Muthanna	1.4 ± 0.12
12	Female	3	Muthanna	0.92 ± 0.1
13	Male	8	Muthanna	0.98 ± 0.16
14	Female	20	Muthanna	1.51 ± 0.17
15	Male	18	Muthanna	1.37 ± 0.19
16	Male	45	Muthanna	1.84 ± 0.14
17	Female	8	Muthanna	1.16 ± 0.12
18	Female	26	Muthanna	2.09 ± 0.21
19	Female	45	Muthanna	1.94 ± 0.25
20	Male	20	Muthanna	1.28 ± 0.15
21	Female	25	Dhi-Qar	1.44 ± 0.18
22	Male	5	Dhi-Qar	1.15 ± 0.26
23	Female	56	Dhi-Qar	2.1 ± 0.17
24	Male	8	Dhi-Qar	1.11 ± 0.08
25	Male	18	Dhi-Qar	1.22 ± 0.17
26	Female	14	Dhi-Qar	1.4 ± 0.21
27	Male	5	Dhi-Qar	0.86 ± 0.15
28	Female	10	Dhi-Qar	1.24 ± 0.19
29	Female	11	Dhi-Qar	1.2 ± 0.41
30	Male	38	Dhi-Qar	1.48 ± 0.17
Mean ± Std Error				1.43 ± 0.07

From Tables 2, 3, the mean value of uranium concentration in blood samples of the leukemia patients group was two times higher than those of the healthy group, and this finding is in agreement with those of other researchers [11, 23].

The independent sample *t* Test confirmed statistically significant difference in the uranium concentration between the leukemia patients and healthy group (*P* < 0.001).

The mean value of uranium concentration in blood samples of the leukemia patients and healthy group in this study is higher than published values with other researchers [10, 11, 22]. The reason behind such results can be attributed to the fact that the area of the current study (southern Iraq) was the center of military activities during the Gulf wars, and the DU was and still an environmental pollution problem because its levels raised after Gulf wars, and the contaminated places haven’t been limited or isolated to stop and avoid the spreading of this radioactive contamination. This explains reasons behind the high concentration in the blood of children born after the Gulf war [24].

Table 4 represents the mean value of uranium concentration in the blood samples of male and female leukemia patients group and healthy group. From this table, the mean value of uranium concentration of male and female leukemia patients group is 2.82 and 2.9 ppb respectively, while the mean value of uranium concentration of male and female healthy group is 1.37 and 1.48 ppb respectively.

**Table 4:** Uranium concentration in blood samples for males and females

	Gender	No. of subjects	Mean ± Std error
Leukemia patients group	Male	14	2.82 ± 0.17
	Female	16	2.9 ± 0.14
Healthy group	Male	14	1.37 ± 0.11
	Female	16	1.48 ± 0.1

The results showed that the average values of uranium concentration for female patients group and healthy group are higher than those for male patients group and healthy group. This is because the total blood volume in females is 4–5 L, while in males is 5–6 L [25].

Results showed no statistically significant difference in the uranium concentration with regard to gender in both groups (*P* > 0.05).

Table 5 illustrates the average of uranium concentration in the blood samples of the leukemia patients group and healthy group as a function of residential place. This table shows that the mean value of uranium concentration in blood samples of leukemia patients group in Basrah, Muthanna and Dhi-Qar is 3.21, 2.77 and 2.61 ppb respectively, while the mean value of uranium concentration in blood samples of healthy group in Basrah, Muthanna and Dhi-Qar is 1.53, 1.45 and 1.32 ppb respectively. It is obvious that the mean value of uranium concentration in the blood samples of the patients group and healthy group in Basrah is higher than those in Muthanna and Dhi-Qar; because Basrah is the region which received the highest amount of DU during the Gulf wars I and II in 1991 and 2003 respectively. Results showed statistically significant correlation with residential area (*P* < 0.05).

**Table 5:** Uranium concentration in the blood samples as a function of residential place

	Governorate	No. of subjects	Mean ± Std error
Leukemia patients group	Basrah	10	3.21 ± 0.17
	Muthanna	10	2.77 ± 0.23
	Dhi-Qar	10	2.61 ± 0.11
Healthy group	Basrah	10	1.53 ± 0.15
	Muthanna	10	1.45 ± 0.12
	Dhi-Qar	10	1.32 ± 0.1

## Conclusion

The results obtained show that uranium concentrations in the blood samples of the leukemia patients group are higher than those of the healthy group. These results show a direct relation between the disease of these patients and the uranium content in the blood.
